# Decomposed and composed effects of economic freedom on economic growth in south Asia

**DOI:** 10.1016/j.heliyon.2023.e13478

**Published:** 2023-02-08

**Authors:** Shabbir Ahmed, Mansoor Mushtaq, Mochammad Fahlevi, Mohammed Aljuaid, Sebastian Saniuk

**Affiliations:** a Department of Economics, Government Islamia Graduate College, Kasur, Pakistan; b Fast School of Management, National University of Computer and Emerging Sciences, Islamabad, Pakistan; c Management Department, BINUS Online Learning, Bina Nusantara University, Jakarta 11480, Indonesia; d Department of Health Administration, College of Business Administration, King Saud University, Saudi Arabia; e Department of Engineering Management and Logistic Systems, Faculty of Economics and Management, University of Zielona Góra, Poland

**Keywords:** Economic freedom, Economic growth, OLS, Random effect, Robust least square

## Abstract

It is frequently asserted that high levels of economic growth are supported by economic freedom. For the period 1995–2021, this study examines the influence of the composed economic freedom index and several subcomponents of economic freedom on the economic growth of four South Asian economies, namely Bangladesh, India, Pakistan, and Sri Lanka. The Ordinary Least Squares, Random Effect Model, and Robust Least Squares approaches are utilized to estimate the composed and decomposed influence of economic freedom on economic growth. Robust Least Squares reflects the robustness of the connection between economic liberty and growth. According to the results of these tests, economic liberty has a strong and favorable stimulus on growth. When the different indicators of economic liberty are evaluated independently, we discovered that the magnitudes of most economic freedom indicators are significant. Conversely, monetary freedom contributes very little to economic expansion. The effects of government spending, public trust, and labor flexibility on economic expansion are hypothetical. The tax load hinders economic expansion in the economies under consideration. Property rights, freedom to do business, trade liberty, investment choice, and financial liberty all have a positive, strong, and sizeable stimulus on economic growth. The decomposed influence of each indicator of economic freedom will help develop policy choices.

## 1 Introduction

In many economies, the lack of economic evolution is one of the most severe economic issues, both currently and historically [1,2]. Nevertheless, why do some countries enjoy rapid economic growth even though others do not, and what factors contribute to economic growth? These are complicated issues that have vexed economists and social scientists since Adam Smith's Day and continue to do so now [3,4]. According to Berggren [5], the imperative subject is “which economic rules and strategies are most beneficial to economic affluence?” Rules and strategies that encourage market-based institutions have been proposed as a sustainable path to long-term economic progress.

Market-based institutions are frequently recognized as necessary for effective resource allocation and economic progress. According to The World Bank [6], these institutions are economic plans, implementation of strategies, and administrations that support market connections and contracts. Their goals are to transmit information effectively, enforce property and agreement rights, and compete vigorously, which affects free market incentives to contribute [7]. According to new growth theories, institutional eminence has an inspiring stimulus on economic growth [4,[8], [9], [10]].

Several empirical kinds of research support the link between institutional preeminence and integrity and socioeconomic prosperity [[11], [12], [13]]. A country with a more advantageous business climate will expand more quickly than a less favorable business environment [14]. One notable line of study that has expanded traction in the last decade emphasizes the importance of economic freedom as a predictor of economic growth [5]. Most studies in this literature demonstrate a favorable relationship between indicators of economic freedom and economic development [5,14].

A country is accessible when there is less government involvement in market-based institutes. People may participate in conjoint and protected agreements. Conferring to Gwartney et al. [15], people obligate economic liberty when the assets they attain without the practice of power, scam, or robbery are sheltered from physical offensives by others. Individuals are allowed to practice, interchange, or trade off their possessions and assets as long as their activities do not interrupt the equivalent honors of others. This explanation makes it evident that economic freedom has various distinct characteristics, all related to the institutions and policies that control countries' economies. Furthermore, economic liberty influences the encouragement configuration in which economic shareholders, stockholders, policymakers, and manufacturers operate. According to this view, economic freedom should serve as a driving force for positive change and growth in the economy, resulting in national prosperity.

Economic freedom is anticipated as an object and a necessary element of social self-esteem, sovereignty, and individual authorization. Economic freedom provides a persistent and accurate strategy for economic advancement and realization [16]. No government assurances seamless liberty to its residents, and those that do allow high levels of freedom differ in their views on which parts are essential [17]. This is consistent with the essence of liberty, which permits people and society to forge their routes to wealth [18]. Many empirical pieces of research have revealed a link between economic freedom and economic progression (e.g., Refs. [[19], [20], [21]]). Some of these studies employed single or two economic freedom indicators, such as property rights, employment freedom, and openness, while others used a variety of economic freedom indices.

Even though multiple indices provide comparable findings, and there might be a strong association between discrete measurements and an inclusive index, the choice of measure is essential. A sole element of economic liberty may not adequately capture the economic and financial status quo. A significantly combined catalog of economic liberty makes drawing policy implications challenging. As a result, it is critical to explore which features of the economic freedom indices are noteworthy for growth and the pathway of these effects. In this article, we divide the standard measure of economic liberty into ten types and test the influence of each type in growth regressions using data from four South Asian nations over 27 years. Using the same sample, we also examine the influence of the overall economic freedom index on economic growth.

### 1.1 Research problem

Individuals have certain economic rights like the choice to work, property control, and use of their labor subject to state laws. The protection of fundamental property rights and the application and effectiveness of the law in enforcing contracts are principally reflected in this minimum state intervention. All other areas of economic flow must be freed from government intervention and allowed to market forces. The qualitative category must be measured, measurable, and comparative to prove that economic liberalization boosts growth. The Heritage Foundation, in collaboration with the Wall Street Journal, has been monitoring the degree of economic liberalism in nearly every nation for more than 20 years. The Index of Economic Freedom reports the state of economic freedom worldwide annually. In order to ascertain the effect of economic freedom on economic growth within four south Asian economies examined from 1995 to 2021, the given Index and its categories will be employed in this study. According to earlier studies, the economic growth of some nations cannot always be accurately predicted by the aggregate Index of economic freedom. We wanted to see if South Asian economies grow when all characteristics of economic freedom are implemented evenly, harmoniously, and fairly or if certain are significant. Given the significant impact of economic freedom on the prosperity of individuals and entire nations, it is seen as vital and necessary to address this issue to enhance society's overall economic advancement.

### 1.2 Objectives of the study

This paper aims to study the effect of economic liberty acquired over and done with the Economic Freedom Index of the Heritage Foundation on the economic growth of south Asian countries. The research will also examine the impact and relative importance of economic liberty's sub-indices on economic growth. This study will determine which component best promotes South Asia's economic growth.

In the remaining division of the article, a literature review is in Section 2. Section 3 specifies the model, Sample section, data sources, and variables. Section 4 explains the methodology, Section 5 performs the empirical finding; Section 6 argues the findings within the scope of related literature and concludes the study.

## 2 Literature review

Many economists and social scientists have confirmed that the freedom to choose and efficient use of resources are crucial components of economic development, if not before Adam Smith. Widespread competition, global trade, and the reliability of properties are solid pillars of economic development [3,22]. Smith [23] explained how the invisible market impacted increased economic prosperity. In 1821, Ricardo advocated for the free market to advance the economy. Friedrich Hayek's alternative interpretation of the monetary theory, formerly popular, is currently seeing a revival. The foundation of neoliberalism is Hayek's book “The Road to Serfdom,” which holds that government economic intervention results in the repression of freedom.

In contrast, freedom is the primary engine of growth [24]. Hayek demonstrated that the government should regiment citizens and take away many of their most valued liberties. It must plan or control the economy in detail and many intellectuals and politicians of the time asked for it. It is believed that free societies have emerged and thrived only because of economic freedom. It is a far more efficient method of controlling the economy than any alternative [15]. Friedman also emphasizes how limiting trade freedom and managing capital flows nullify exchange profits, lowering the output and yields of economic activities and impeding investment incentives. The classical tradition of freedom, defined by Mises [25], is “the liberal doctrine of the harmony of the rightly understood interests of all members of a free society founded on the principle of private ownership of the means of production.” Mises [25] presented the theoretical and practical arguments for freedom in this classical tradition. According to Mises, the principles of liberalism are based on a knowledge and appreciation of private property, social cooperation, the concept of freedom, ethics and morality, democracy, and the proper function of government. Mises contrasts liberalism with other potential social order systems like socialism, communism, and fascism in this work as well.

Furthermore, according to Rothbard [26], civilization depends on these disparities since humans are intrinsically different from one another due to biodiversity and history. The division of labor, the foundation of a developed economic system, arises from the reality that people have various skills. Many institutional variables, including property rights, justice, women's empowerment, and political and social stability, are linked to economic growth. According to Goldsmith [27], less developed nations that uphold economic and political independence guarantee better social and economic welfare. In less developed economies, less economic liberty causes unemployment and insufficiency [28,29].

With particular emphasis on economic freedom and the origins of limited government in political fragmentation and interstate rivalry, Weede [30] explains the disparate economic performance of Asia's economic juggernauts and the West. He also examines numerous reasons why China may outperform India as both countries' economies grew faster than the world's and began their catch-up growth spurts. Technological advancement, research and development, cultural diversity, individual freedom, property rights, agricultural reforms, affordable manufacturing, and trade freedom stood out among these elements. He also discovered that the West surpassed China and India due to a lack of political and civic liberty. Economic freedom is essential for both prosperity and political stability.

The primary factor for the extreme disparities in income and well-being between countries is defined by renowned German educational and developmental psychologist Rindermann [31] in his book “Cognitive Capitalism, Human capital, and the wellbeing of nations.” His line of reasoning is as follows: In the modern economy, which is becoming increasingly more complicated and knowledge-based, cognitive competence—which includes intelligence, knowledge, and the capacity to use this knowledge—is becoming more and more crucial. Therefore, having a higher level of cognitive ability will generally result in more remarkable economic growth and well-being: directly, because a more qualified and skilled labor force and a more innovative and creative intellectual climate will result; indirectly, because it will have a positive impact on the standard of institutions (such as the government, legal system, or judicial system). Hoppe [32] examines the traditional liberal view of the possibility of limited government and advocates for the arrangement of libertarianism and conservatism as associated with shared objectives. He argues that insurance companies, the business community, and the trade sector have an appropriate place in producing goods and services in a free market.

Many empirical studies also show the relationship between different elements of economic freedom and economic growth in various countries. Like, Barro [11] uses the black-market premium on foreign reserves as a substitution for governmental market inefficiencies. Its magnitude in a growth regression derived for around 100 nations is strongly adverse, implying that market falsifications harm economic growth. However, one can question if the black-market premium is a reliable predictor of economic sovereignty. Scully and Slottje [19] and De Vanssay and Spindler [18] discovered a positive association between economic growth and this measure of economic liberty utilizing a classification of economic liberty established by them.

Torstensson [16] examined the influence of economic freedom on economic evolution utilizing data from 68 nations from 1976 to 1985. This paper looks at two issues of property rights. The first issue attempts to determine the extent to which the state controls the property, while the other issue attempt to determine if the person is secure from arbitrary confiscation of growth.

Nelson and Singh [33] analyze the link between economic progression and political liberty using economic liberty as a control predictor. Their research spans the years 1970–1989 and covers 67 developing nations. Price permanency, government magnitude, unfair taxes, and trade restrictions are used to gauge economic freedom. They conclude that economic freedom has a strong beneficial effect on economic progression. Gwartney et al. [15] arrange for supplementary evidence on the influence of economic authorization on growth. Data from the Fraser Institute for 82 nations from 1975 to 1995 show that freedom has a favorable effect even when human and physical resources and demography are considered.

Wu and Davis [34] used contingency analyses for a panel of around 100 nations from 1975 to 1992 to confirm the positive association between freedom and growth. Heckelman [35] uses the Heritage Foundation's disaggregated Index to test a short-run causation analysis between freedom and growth. It has been discovered that practically all components, except intervention, trade policy, and taxes, positively promote growth. In his other study with Stroup [36], he discovered that five of the fourteen components examined by the Fraser Institute's Index negatively influence growth. Carlsson and Lundstrom [37] disaggregate the Fraser Institute's economic Index for 74 nations and, using a standard growth model, conclude that not only legal framework and flexibility to use other currencies have a positive influence on growth.

Bengoa and Sanchez-Robles [38] examine the link concerning economic liberty, FDI, and development in 18 Latin American countries from 1970 to 1999. They conducted a panel data analysis to examine the relationship between economic liberty, FDI, and economic growth. Conferring to their findings, economic liberty and FDI are positively and strongly connected with development. Chheng [39] examines the combined effect of gross fixed capital and economic sovereignty on economic expansion for a panel of fifty economies from 1981 to 2000 using White's heteroscedasticity-consistent matrix tests. He discovers that economies that have proved economic liberty and sustained physical capital have had better levels of economic expansion.

Averre [40] explores the relationship between economic sovereignty and economic development in three European Union samples. He discovers a strong and statistically significant link between economic sovereignty and economic development. Justesen [41] utilizes Granger-causality tests and discovers that economic independence in government magnitude and governing strategies have a substantial and progressive influence on growth. However, other aspects of economic independence do not. Saribas [42] investigates the liaison between economic growth and freedom in 49 countries from 1995 to 2004. His findings indicate that economic growth is inversely related to economic freedom.

Garrett and Rhine [43] encompass economic growth models by reconnoitering the influence of economic freedom on employment progression in the United States. They claim that economic freedom has a worthy and significant impact on employment progression. Pourshahabi et al. [44]use panel data analysis to examine the link between FDI, human capital, economic freedom, and growth in OECD nations from 1997 to 2007. According to their findings, greater economic freedom can, directly and indirectly, boost economic growth. It can indirectly boost economic growth by encouraging incentives, productive effort, and resource efficiency.

Pourshahabi et al. [44] examine the stimulus of economic independence on economic expansion in OPEC from 2000 to 2009. They proposed that an all-inclusive indicator of economic independence was confidently and strongly linked to economic expansion. Kilic and Arica [45] investigate the relationship between economic freedom, price instability, and economic accomplishment in twenty-three emerging nations. They investigate whether economic liberty has a positive and significant impact on the economic performance of emerging economies. On the other hand, price instability had a negative and significant influence on economic accomplishment. The inclusive effect of the freedom index on economic growth is promising and remarkable. While the individual influence of some components, such as government size and corporate freedoms, is negligible. The remaining factors have a positive and statistically noteworthy influence on economic accomplishment.

Kesikoglu [46] studies the linkage between economic freedom and economic growth in 11 transition economies from 1996 to 2013. Only Albania and the Czech Republic show a causative liaison between growth and economic freedom. In contrast, the other 11 transition economies show no such relationship. Haydaroglu [47] explores the effects of foreign investment and economic independence on the growth of five economies: Brazil, Russia, India, China, and South Africa. He investigates how the overall magnitude of economic liberty has had a positive and considerable impact on economic growth. Ahmad [48] investigates the link between economic independence and wealth disparities in 115 nations worldwide. They used the GMM estimate approach and discovered that in the presence of a democratic government, economic freedom had a progressive impact on income variations.

Medina-Moral and Montes-Gan [49] investigate the link between economic freedom, governance, and global economic progress. To conduct empirical research, they utilized panel data and a probit model. The findings show that economic freedom is the most significant institution at all levels of development. Governance was also shown to be crucial, but only in nations in the middle stage of development. Tran [50] considers the stimulus of economic sovereignty on ASEAN economic growth and realizes that greater economic sovereignty and choice of labor lead to more remarkable economic growth.

Ahmed and Ahmad [51] investigate the influence of economic and political freedom on Asian nations' economic growth. Using panel data from 1995 to 2018, they discover that both types of freedom have a favorable and significant stimulus on economic growth. Ahmed and Ahmad [52] recently investigated the effect of economic liberty on employment levels in Asian countries. They find a positive association between economic liberty and employment level in Asia.

The research reviewed examined the link between economic freedom and growth or per capita income. Some of these studies employed one or two economic freedom indicators, such as government size or free trade. In contrast, others used aggregate economic freedom indices. Although several discovered a favorable impact of freedom on growth, the choice of measure and element of economic freedom remains essential and imperative. A single component does not adequately redirect the economic situation. An exceedingly accumulated directory of freedom makes drawing policy implications interesting. As a result, to gain a subterranean consideration of the situation, we included all components and the aggregate Index of economic freedom in our model.

## 3 Model

The general form of the model can be written as

(1) $$lnRGDP_{it}=\beta_{0}+\beta_{1}L_{it}+\beta_{2}K_{it}+\beta_{3}T_{it}+\beta_{4}X_{it}+\mu$$

Where in equation (1) β_0_ = Intercept. *i* = Cross-section dimensions. *t* = Time-series dimensions. $U_{it}$ = Error term ${lnRGDP}_{it}$ = Natural log of real GDP at chained PPPs (in mil. 2017US$). Data was taken from Penn world Table S10.0. *L*_it_ = The Labor force is measured by the natural log of persons engaged (in millions). Data was taken from Penn world Table S10.0.$K_{it}=$ Physical capital is measured by the natural log of Capital stock at current PPPs (in mil. 2017US$). Data was taken from Penn world Table S10.0. $T_{it}=$ Technological Improvement is measured by the natural log of total Patent applications (residents plus nonresidents). Data was taken from world Development Indicators (WDI) 2021.$X_{it}=$ Economic freedom index and its components.

### 3.1 Sample selection and data sources

In the first phase of country selection, the sample encompassed Asia and North African economies due to conjoint cultural, ethnic, and geographical physiognomies. The Asia region covers the world's prevalent geographic area, surrounding almost 48 countries that stretch from Japan, Southeast Asia, and across India to the Caspian Basin in the West. Asia's overall economic freedom score of 61.1 is just below the world average in the 2021 Index. The region has continued to lead worldwide economic growth, expanding by an average annual rate of about 6% over the past five years. As Asia combines different regions, cultures, norms, natural endowment, and income groups, we further classify and minimize the sample size.

We exclude North Africa and all oil-producing and exporting economies such as Saudi Arabia, Azerbaijan, Kuwait, Russia, Qatar, Oman, United Arab Emirates, Iran, and Yemen, etc. In the subsequent phase, we also excluded East Asian countries such as China, Japan, Mongolia, North Korea, South Korea, and Taiwan due to their particular geographical and ethnocultural features. Eleven countries of Southeast Asia, Brunei, Burma, Cambodia, Timor-Leste, Indonesia, Laos, Malaysia, the Philippines, Singapore, Thailand, and Vietnam, are not included in the sample due to the composed of impressive diversity in religion, culture, and history. Heritage Foundation also categorized the Asian Region into three broad categories: high-free, moderate-free, and less-free economies. Armenia, Cyprus, Indonesia, Japan, Malaysia, Singapore, South Korea, and Thailand were not included due to high rankings regarding economic freedom, property rights, financial liberty, and economic growth. Afghanistan, North Korea, Nepal, Laos, Timor-Leste, and Turkmenistan were not included due to less free economies.

In the consequent step, we were left over with seven South Asian economies. The complete empirical data on Bhutan, Nepal, and Maldives was unavailable. So, we select only four south Asian economies, Bangladesh, India, Pakistan, and Sri Lanka, for empirical analysis. That region has specific common social and economic characteristics. This region's economic growth, educational attainments, customs, and culture are conjoint. The economic freedom score of the four economies is much close to each other as Bangladesh, India, Pakistan, and Sri Lanka have 56.5, 56.5, 51.7, and 55.7 scores for economic freedom, respectively. The arrangement of property rights, government integrity, tax burden, financial freedom, investment liberty, and trade freedom is also near to each other in these economies. One of the fastest-growing regions in the globe, South Asia is expected to have a GDP growth rate of 7.1% by 2022, up from an average of 5–7% during the past two decades. After the global financial disasters of 2008, the associate countries need to provide economic liberty. Governments must be vigilant about economic sovereignty to fortify and resume their economies. Since the early 1990s, South Asian nations have seen economic, political, and social liberation from their former conventional economies. (Khan, 2012). With this freedom, these nations have made significant progress, with an average annual economic growth rate of 5.5% over the past 20 years. The pursuit of stable economic growth is a goal for South Asian nations. These nations' varied responses to economic liberalization between 1995 and 2021 show how seriously they regard these challenges. The political situation in south Asian economies varies slightly. Bangladesh, Pakistan, and Sri Lanka have equal political stability and civil freedom. However, India has a strong democracy, upright political rights, and worthy civil freedom. Nevertheless, every nation in South Asia supports a democratic system of government. South Asian nations represent diversity in every way while still forming a geopolitical area.

Data of all variables were taken from Heritage Foundation, World Development Indicators (WDI) 2021and, Penn world Table S10.0. We used panel data from four south Asian Economies, Bangladesh, India, Pakistan, and Sri Lanka, for the period 1995 to 2021, which combines country and time dimensions. So, every country has 27 observations, and 108 were included in the sample. The economic freedom Index and its sub-components are explained below.

### 3.2 Economic freedom (EF)

The Index of Economic Freedom is based on examining 12 economic freedom indicators. The rights of property, jurisdictive efficiency, government truthfulness, tax liability, government outlay, fiscal health, business liberty, labor independence, monetary sovereignty, trade liberty, investment liberty, and financial freedom are among these components. The Index assesses 12 aspects of economic freedom, each of which is evaluated on a scale of 0–100. Scores closer to 100 imply more freedom, while scores closer to 0 indicate less. Scores on these 12 indicators of economic freedom are in the same way weighted and summed to generate an overall economic freedom score for each economy. In our study, we do not include only two components, judicial effectiveness, and fiscal health, due to the non-availability of data.

### 3.3 Property rights (PR)

The property rights component evaluates how far the national legal system allows individuals to acquire, own, and use private property and how well the government enforces explicit law.

### 3.4 Government integrity (GI)

Corruption impairs economic freedom by instilling fear and compulsion in economic transactions. The systemic corruptions of governmental institutions and the decision-making of behaviors such as bribery, extortion, nepotism, sponsorship, embezzlement, and bribery are of particular concern. The lack of government integrity in such actions undermines public trust and financial vigor by raising business costs. This component's score is calculated from the average score of the three sub-factors listed below, equally weighted. Risk of Bribery, Perceptions of Corruption, and Managing Corruption, for example, includes the “conquest” of the state by private and elite interests.

### 3.5 Tax burden (TB)

The tax burden represents marginal tax rates on both personal and corporate income and the total tax level as a percentage of gross domestic product (GDP), including direct and indirect taxes collected by governments at all levels. It is a compound index that will be utilized.

### 3.6 Government spending (GS)

The government spending component includes government outlay and transfer payments related to different qualifying programs, representing the cost imposed by government expenses. The scale used to evaluate government spending is non-linear. This indicates that the penalty will be minor if government expenditure is near zero. However, if government spending surpasses 30% of GDP, the penalty will be twice (for example, spending four times less). Merely high government expenditure (58% or more of GDP) is classified as zero.

### 3.7 Business freedom (BF)

The aspect of business freedom assesses the extent to which national rules and the infrastructural environment impede a company's effective functioning. Quantitative ratings are generated from various elements influencing the ease of founding, operating, and terminating a firm. The business freedom score for each nation ranges from 0 to 100, with 100 signifying the most liberal business climate. Scores are determined by four sub-factors, each of which is equally weighted. These are access to energy resources, environmental hazards in business, regulatory characteristics, and women's economic inclusion.

### 3.8 Labor freedom (LF)

The element of labor freedom is a quantifiable measure that includes different parts of the national labor market's legal and regulatory structure. It includes minimum wage regulation, the right to organize, regulations prohibiting layoffs, severance obligations, and quantifiable regulatory restrictions. Employment and hours of employment and also participation and labor productivity, as measures of labor market employment opportunities.

### 3.9 Monetary freedom (MF)

Monetary freedom integrates inflation measurement with evaluating different government operations that distort pricing. In the absence of microeconomic interference, price stability is optimal for a free market. The monetary freedom factor score comprises two sub-factors: a qualitative evaluation of the three-year weighted annual inflation rate and the level of government price fixing through immediate supervision or subsidies.

### 3.10 Trade freedom (TF)

Trade freedom is a combined measure of the tariff and non-tariff barriers to goods and services, imports and exports. The trade freedom score is calculated using two factors:

The trade-weighted average tariff rate and a qualitative assessment of non-tariff barriers (NTBs).

### 3.11 Investment freedom (IF)

There are no constraints on the movement of investment in an economically free country. Individuals and corporations can shift resources to other countries and territories. In the Index, such an ideal country would obtain a score of 100 for the factor of investment freedom.

### 3.12 Financial freedom (FF)

Financial freedom is a measure of authorization from government control and interference in the financial industry, over and above an indicator of banking efficiency. The Index measures an economy's financial authorization in five zones: the breadth of state regulation on financial sector services, the scope of government participation in banks and other financial enterprises through direct and indirect ownership, the influence of government on lending and capital and financial markets foreign competition and openness to development.

In our econometric analysis, the underlying data have been standardized so that the variances of dependent and independent variables are equal to 1. Therefore, standardized coefficients are unit less and refer to how many standard deviations a dependent variable will change per standard deviation increase in the predictor variable. Using standardized effects means that the effect (beta) of variable A (predictor) on target variable Z (criterion) can be compared to the effect of variable B (predictor) on target variable Z (criterion). The larger the value, the larger the statistical effect. Standardizing coefficients means that we can compare the relative importance of each coefficient in a regression model [53]. The following equations (2), (3), (4), (5), (6), (7), (8), (9), (10), (11) are used to see the impact of sub-component of economic freedom index on economic growth.

(2) lnRGDP_1it_=β_01_+β_11_L_it_+β_21_K_it_+β_31_T_it_+β_41_PR_it_+

(3) lnRGDP_2it_= β_02_+β_12_L_it_+β_22_K_it_+β_32_T_it_+β_42_GI_it_+

(4) lnRGDP_3it_ = β_03_+β_13_L_it_+β_23_K_it_+β_33_T_it_+β_43_TB_it_+μ

(5) lnRGDP_4it_ = β_04_+β_14_L_it_+β_24_K_it_+β_34_T_it_+β_44_GS_it_+μ

(6) lnRGDP_5it_ = β_05_+β_15_L_it_+β_25_K_it_+β_35_T_it_+β_45_BF_it_+μ

(7) lnRGDP_6it_ = β_06_+β_16_L_it_+β_26_K_it_+β_36_T_it_+β_46_LF_it_+μ

(8) $$lnRGDP\_it=\alpha \_0+\alpha \_1\;L\_it+\alpha \_2\;K\_it+\alpha \_3\;T\_it+\alpha \_4\;LF\_it+U\_it\;lnRGDP_{7it}=\beta_{07}+\beta_{171}L_{it}+\beta_{27}K_{it}+\beta_{37}T_{it}+\beta_{47}MF_{it}+\mu$$

(9) $$lnRGDP\_it=\alpha \_0+\alpha \_1\;L\_it+\alpha \_2\;K\_it+\alpha \_3\;T\_it+\alpha \_4\;LF\_it+U\_it\;lnRGDP_{8it}=\beta_{08}+\beta_{18}L_{it}+\beta_{28}K_{it}+\beta_{38}T_{it}+\beta_{48}TF_{it}+\mu$$

(10) $$lnRGDP\_it=\alpha \_0+\alpha \_1\;L\_it+\alpha \_2\;K\_it+\alpha \_3\;T\_it+\alpha \_4\;LF\_it+U\_it\;lnRGDP_{9it}=\beta_{09}+\beta_{19}L_{it}+\beta_{29}K_{it}+\beta_{39}T_{it}+\beta_{49}IF_{it}+\mu$$

(11) $$lnRGDP\_it=\alpha \_0+\alpha \_1\;L\_it+\alpha \_2\;K\_it+\alpha \_3\;T\_it+\alpha \_4\;LF\_it+U\_it\;lnRGDP_{10it}=\beta_{10}+\beta_{20}L_{it}+\beta_{30}K_{it}+\beta_{40}T_{it}+\beta_{50}FF_{it}+\mu$$

We will also analyze the impact of overall index of economic freedom on real GDP growth in equation (12).

(12) lnRGDP_11it_ = β_11_+β_21_L_it_+β_31_K_it_+β_41_T_it_+β_51_EF_it_+μ

## 4 Methodology

We have estimated all regressions with three differing econometric techniques OLS, Random Effect Model, and Robust Least square.

### 4.1 Ordinary least square (OLS)

Ordinary least squares (OLS) is a linear least squaresmethod for approximating the unidentified parametersin a linear regressionmodel. OLS chooses the parameters of a linear function of a set of explanatory variables by the code of least squares. Minimizing the sum of the squares of the differences between the observed dependent variable, e.g., values of the observed variable are given dataset and those predicted by the linear function of the independent variable.

### 4.2 Random Effect Model

If we assume that the undetected country-specific impacts are not correlated, then Random Effect Model is used which is explained in equation (13)

(13) $$Y_{it}=K_{it}\;\psi +E\;\left[W_{i}\;\beta \right]+\left\{W_{i}\;\alpha \;–\;E\;\left[W_{I}\;\beta \right]\right\}+\varepsilon_{it}=K_{it}\;\psi +\beta +\mu_{i}+\varepsilon_{it}$$

It is a Linear Regression Model with a composite error term; this is the random effects approach, which indicates that *μ*_*i*_ is a country-specific random factor like *ε*_*it*_.

### 4.3 Robust least square

If its underlying assumptions are correct, the conventional regression method is the most effective approach, such as ordinary least square estimates (OLS). If some of these assumptions are not fulfilled, it will give misleading results. Thus, ordinary least squares are said to be not robust to violations of their assumptions. Moreover, Ordinary least square (OLS) estimates in multiple regressions are severely affected by outliers, non-normality, multicollinearity, and missing data. The influence of outliers, OLS regression, will give biased results to overcome this issue. Robust regression overcomes the influence of extreme observations. M Estimates define a general function of residuals *H* ($\varepsilon_{i}$), then minimizing *S* = Ʃ *H* ($\varepsilon_{i}$). For OLS *H* ($\varepsilon_{i}$) = ${\varepsilon_{i}}^{2}$. The properties we want for the function *H*; are always non-negative = *H* ($\varepsilon_{i}$) ≥ 0, *H* (0) = 0, symmetric, *H* ($-\varepsilon_{i}$) = *H* ($\varepsilon_{i}$), monotonic: if ǀ $\varepsilon_{i}$ ǀ > ǀ $\varepsilon_{j}$ ǀ then *H* ($\varepsilon_{i}$) ˃*H* ($\varepsilon_{j}$). For least–squares regressions: *S* = Ʃ ${\varepsilon_{i}}^{2}$ in equations (14), (15), (16).

Diff. w.r.t. parameter and keep equal to zero

(14) $$\frac{\partial S}{\partial \beta_{k}}=0\to \sum_{i=1}^{i=0}{\;\varepsilon }_{i}x_{it}=0$$

For M − Estimators,

(15) $$\frac{\partial S}{\partial \beta_{k}}=0\to \sum_{i=1}^{i=0}\;\frac{\partial H}{\partial \varepsilon_{i}}x_{ki}=0$$

Define weight as

(16) $$w_{i}=\frac{1}{\varepsilon_{i}}\frac{\partial H}{\partial \varepsilon_{i}}$$

#### 4.3.1 Giving

(17) $$\sum_{i=1}^{n}\frac{\partial H}{\partial \varepsilon_{i}}x_{ki}=0=\sum_{i=1}^{n}\;\frac{\partial H}{\partial \varepsilon_{i}}w_{i}\varepsilon_{i}x_{ki}$$

Nevertheless, this is just a weighted linear regression in equation (17). Guess the weight and fit, then calculate the residuals. Use those residuals to calculate the new weights. Repeat until convergence called interactively reweighted Least Squares.

## 5 Econometric findings

In the first stage of this section, we examined the decomposed effects of economic freedom on the economic growth of south Asia by utilizing the Ordinary Least Square (OLS) Method in Table 1 Ten different regressions were estimated by including the alternative components of economic freedom. In all regression, our supporting variables, such as labor force, physical capital, and technological advancement, have significant and positive impacts on economic growth. In decomposed analysis, the results of OLS provide a mix of impacts on economic growth. The standardized beta coefficient of OLS compares the power of the influence of each componentto economic growth. Business freedom, trade liberty, investment rights, and financial independence have positive and significant impacts on the economic progress of South Asian economies. Monetary freedom has an inconclusive impact, while Tax burden is considered harmful to economic progress. Standardizing coefficients define the relative importance of business freedom, trade freedom, investment freedom, and financial independence over monetary liberty, labor freedom, and property rights.

**Table 1:** OLS Results (Decomposed Analysis) Dependent Variable: lnRGDP.

	1	2	3	4	5	6	7	8	9	10
C	1.07 (8.29)*	1.11 (8.77)*	1.08 (8.48)*	−0.001 (−0.081)	1.08 (8.58)*	1.09 (9.14)*	1.09 (8.41)*	1.06 (8.41)*	1.13 (9.46)*	1.17 (1.02)
lnL	0.191 (5.934)*	0.141 (3.566)*	0.163 (4.651)*	0.201 (6.302)*	0.246 (6.542)*	0.179 (6.114)*	0.19 (5.91)*	0.273 (5.78)*	0.138 (4.39)*	0.211 (7.42)*
lnK	0.49 (9.0)*	0.47 (8.97)*	0.50 (9.44)*	0.48 (8.61)*	0.48 (9.51)*	0.51 (10.49)*	0.48 (8.59)*	0.44 (8.06)*	0.54 (10.81)*	0.57 (11.62)*
lnT	0.340 (7.161)*	0.416 (7.894)*	0.353 (8.635)*	0.336 (7.461)*	0.313 (7.455)*	0.314 (8.141)*	0.349 (8.08)*	0.316 (7.47)*	0.348 (9.18)*	0.259 (6.44)*
PR	0.0053 (0.341)									
GI		−0.040 (−2.021)**								
TB			−0.023 (−1.60)							
GS				−0.028 (−2.11)**						
BF					0.047 (2.633)*					
LF						−0.055 (−4.36)*				
MF							−0.0019 (−0.14)			
TF								0.047 (2.35)**		
IF									0.056 (4.32)*	
FF										0.073 (5.33)*
Obs	108	108	108	108	108	108	108	108	108	108

Note: *, **, and *** specify the significance level at 1%, 5%, and 10%. While t values are in parentheses. Whereas, Constant (C), natural log of Labour force (lnL), natural log of capital stock (lnK), Technological Improvement measured by the natural log of total Patent applications (lnT), Property Rights (PR), Government Integrity (GI), Tax Burden (TB), Government Spending (GS), Business Freedom (BF), Labor Freedom (LF), Monetary Freedom (MF), Trade Freedom (TF), Investment Freedom (IF), Financial Freedom (FF).

### 5.1 Hausman test

Hausman's [54] test has been applied to see either the Fixed Effect Model is suitable or the Random Effect Model. The null hypothesis is that the Random Effect Model is appropriate for the estimation of the model whereas the alternate hypothesis is that the Fixed Effect Model is applicable and Table 2 presents the results of Hausman test. Hausman [54] test rejected the fixed effect model application and suggested adopting the Random Effect Model (see Table 3).

**Table 2:** Hausman test.

Specifications	$X^{2}$ statistic	$X^{2}$ D.F	Prob.
1	5.842848	4	0.2112
2	3.543594	4	0.4713
3	4.040833	4	0.4005
4	1.953802	4	0.7443
5	4.659701	4	0.3240
6	2.905926	4	0.5737
7	5.062251	4	0.2810
8	6.354458	4	0.1742
9	5.690996	4	0.1693
10	2.168740	4	0.1302

**Table 3:** Random Effect Model (Decomposed Analysis) Dependent Variable: lnRGDP.

	1	2	3	4	5	6	7	8	9	10
C	1.07 (7.45)*	1.11 (7.41)*	1.08 (7.57)*	−0.001 (−0.07)	1.06 (7.59)*	1.09 (8.1)*	1.08 (7.45)*	5.79 (7.18)*	5.81 (7.70)*	5.80 (8.61)*
lnL	0.19 (5.335)*	0.14 (3.17)*	0.16 (4.15)*	0.20 (5.53)*	0.25 (5.86)*	0.17 (5.42)*	0.19 (5.29)*	0.15 (5.22)*	0.08 (4.02)*	0.12 (7.31)*
lnK	0.48 (8.097)*	0.46 (7.98)*	0.5 (8.43)*	0.47 (7.55)*	0.48 (8.52)*	0.51 (9.31)*	0.47 (7.7)*	0.25 (7.29)*	0.31 (9.9)*	0.33 (11.45)*
lnT	0.34 ( 6.438)*	0.42 (7.03)*	0.35 (7.71)*	0.34 (6.55)*	0.31 (6.68)*	0.31 (7.21)*	0.34 (7.24)*	0.18 (6.75)*	0.20 (8.4)*	0.14 (6.35)*
PR	0.005 (0.305)									
GI		−0.04 (−1.8)***								
TB			−0.023 (−1.42)							
GS				−0.029 (−1.85)***						
BF					0.046 (2.36)**					
LF						−0.055 (−3.87)*				
MF							−0.001 (−0.13)			
TF								0.027 (2.13)**		
IF									0.032 (3.95)*	
FF										0.043 (5.25)*
Obs	108	108	108	108	108	108	108	108	108	108

Note: *, **, and *** designate the significance level at 1%, 5%, and 10%; t-values are in parentheses.Whereas, Constant (C), natural log of Labour force (lnL), natural log of capital stock (lnK), Technological Improvement measured by the natural log of total Patent applications (lnT), Property Rights (PR), Government Integrity (GI), Tax Burden (TB), Government Spending (GS), Business Freedom (BF), Labor Freedom (LF), Monetary Freedom (MF), Trade Freedom (TF), Investment Freedom (IF), Financial Freedom (FF).

We estimate ten growth regressions using three distinct econometric methodologies in our decomposed analysis. Property rights have a favorable influence on real GDP in three models. Then again, they significantly influence only the Random Effect Model and Robust Least Squares. The influence of government integrity on real GDP is inconclusive. When we estimate the model with OLS and Random effects, it has an undesirable and substantial impact.

In contrast, it has a progressive and substantial impact when we estimate using Robust Least Square. The tax burden has a destructive and significant effect on real GDP. Tax burden hinders all economic, financial, and business activities. Government disbursement, like government integrity, has an equivocal influence on real GDP. Accordingly, no work has been prepared to fix an ideal level of government expenditure. The appropriate expanse will differ from nation to nation based on features such as norms, natural features, and level of financial enlargement. As the public sector grew, government spending became inevitable. It unavoidably leads to the mishandling of resources and loss of economic efficiency. Excessive government spending, which creates persistent budget insufficiencies and the addition of public debt, has been identified as one of the utmost impediments to economic affluence. In three models, the standardized beta coefficients explain business freedom's relative significance and considerable influence on real GDP. Surprisingly, in three econometric outcomes, labor sovereignty has a destructive influence on real GDP. Monetary freedom has a minute effect on real GDP. According to three estimated models, trade freedom has a beneficial influence on real GDP. Businesses increasingly seek the policy of trade freedom in light of the expansion of global supply networks and cross-border manufacturing progressions. Government actions that cause insecurity about a forthcoming line of work may harm trade choice that encompasses further than their instant financial and economic impact. Investment freedom, like trade freedom, has a positive and considerable influence on real GDP. The results of robust least squares indicate the robustness of our coefficients also.

An unrestricted and vast investment maximizes innovative approaches and creates inducements for improved economic doings, efficiency, and job formation. The social order and the sectors that take the business threat to increase turnover benefit from such a situation; hence, investment liberty encourages innovation and perseverance by promoting transparency and equity. Capital mobility constraints, both local and international, impede effective resource allocation and lower productivity, distorting economic policymaking. Cross-boundary investment confines can limit resource influxes and outflows together, diminishing markets and reducing chances for growth. Investment can flow to its best purposes in an environment where people and corporations can choose where and how to finance. State action to redirect the flow of capital and limit choice is an imposition on the freedom of both the investor and the person seeking capital—the more a nation's investment limitations, the lesser the degree of entrepreneurial accomplishment.

Finally, financial independence has a largely beneficial influence on real GDP. An accessible and efficient formal financial system declares that consumers and firms can access a miscellaneous assortment of funds, loans, disbursement, and outlay facilities. By expanding funding options and encouraging entrepreneurship, the unfettered banking system competes to provide the most efficient financial intermediation between families, firms, investors, and entrepreneurs. Aside from assuring financial market openness and integrity, government banking and financial regulation can stifle efficiency, raise corporate funding costs, and limit competition. When the government intervenes in the stock market, it contradicts the decisions of millions of individuals by interfering with capital pricing, which is the most crucial function of the market economy.

In case of composed analysis, Hausman test rejected the application of fixed effect approach in Table 5 (see Table 4). Economic Freedom Index has optimistic and noteworthy impact on real GDP in three models in Table 6. The economies of south Asia can grow further if they improve their economic freedom. The findings of the affirmative impact of economic freedom on economic growth are consistent with Compton et al. [55]. They examined economic liberty's favorable and noteworthy impact on economic growth in the United States. The relationship between economic liberalization and economic growth in five South Asian economies from 1995 to 2007 was examined by Mahmood et al. [56]. They believed that economic advancement and economic liberty were positively correlated. Paakkonen [57] examined the relationship between economic freedom and development in 25 transition economies. He tested the idea that more vital institutions, as seen in terms of economic autonomy, led to development. Using data from 1995 to 2013, Haydaroglu [47] examined the effects of foreign investment and economic sovereignty on the growth of five economies: China, India, Brazil, South Africa, and Russia. He discovered that the total level of economic liberty has a positive and vital impact on economic growth. Ahmed and Ahmad [51] consider the effect of economic and political liberty on Asian nations' economic progress. They realize that both kinds of liberty have an advantageous and noteworthy effect on economic growth. Ahmed and Ahmad [52] also investigated the effect of economic liberty on employment generation in Asian countries. They found a constructive connotation between economic freedom and employment generation in Asian economies.

**Table 4:** Robust Least Square (Decomposed Analysis) Dependent Variable: lnRGDP.

	1	2	3	4	5	6	7	8	9	10
C	−0.076 (−12.75)*	−0.09 (−15.35)*	−0.08 (−13.38)*	−0.083 (−13.9)*	−0.02 (1.8)***	−0.087 (−13.9)*	−0.041 (−3.55)*	5.74 (17.21)*	5.74 (16.31)*	−0.08 (−14.07)*
lnL	0.13 (8.88)*	0.12 (6.31)*	0.067 (4.035)*	0.089 (5.99)*	0.21 (5.59)*	0.08 (5.67)*	0.15 (5.05)*	0.071 (5.68)*	0.047 (5.12)*	0.102 (7.21)*
lnK	0.67 (26.64)*	0.64 (26.28)*	0.62 (25.26)*	0.59 (22.73)*	0.49 (9.4)*	0.6 (23.9)*	0.49 (9.93)*	0.33 (23.01)*	0.33 (23.03)*	0.65 (26.32)*
lnTech	0.21 (9.65)*	0.27 (10.77)*	0.34 (17.89)*	0.35 (16.78)*	0.34 (7.87)*	0.34 (16.87)*	0.38 (9.99)*	0.19 (17.38)*	0.21 (18.85)*	0.28 (14.14)*
PR	0.05 (7.13)*									
GI		0.032 (3.52)*								
TB			−0.04 (−5.97)*							
GS				0.007 (1.11)						
BF					0.044 (2.44)*					
LF						0.015 (2.32)**				
MF							−0.006 (−0.48)			
TF								0.014 (2.81)*		
IF									0.011 (2.86)*	
FF										0.032 (4.75)*
Obs	108	108	108	108	108	108	108	108	108	108

Note: *, **, and *** point out the significance level at 1%, 5%, and 10%. Whereas t-values are in parentheses. Whereas, Constant (C), natural log of Labour force (lnL), natural log of capital stock (lnK), Technological Improvement measured by the natural log of total Patent applications (lnT), Property Rights (PR), Government Integrity (GI), Tax Burden (TB), Government Spending (GS), Business Freedom (BF), Labor Freedom (LF), Monetary Freedom (MF), Trade Freedom (TF), Investment Freedom (IF), Financial Freedom (FF).

**Table 5:** Hausman test composed analysis.

$X^{2}$ statistic	$X^{2}$ D.F	Prob.
4.764	4	0.4460

**Table 6:** Dependent Variable: lnRGDP.

	OLS	Random Effect	Robust Least Square
C	1.05 (8.47)*	0.263 (6.109)*	−0.088 (−19.05)*
lnL	0.263 (6.609)*	0.487 (9.002)*	0.148 (9.978)*
lnK	0.487 (9.737)*	0.289 (5.996)*	0.632 (33.79)*
lnTech	0.289 (6.486)*	0.055 (2.674)*	0.267 (16.03)*
EF	0.055 (2.893)*	1.05 (7.82)*	0.049 (6.88)*
Obs	108	108	108

Note: *, **, and *** show significance levels at 1%, 5%, and 10%. While t-values are in parentheses. Whereas, Constant (C), natural log of Labour force (lnL), natural log of capital stock (lnK), Technological Improvement measured by the natural log of total Patent applications (lnT), Economic Freedom Index (EF).

## 6 Conclusion

Several studies have examined economic freedom's effect on growth [58]. However, due to the influence of numerous components of economic freedom on economic growth, the research on the connection between economic freedom and economic growth is equivocal [28]. Some of these studies focused on only single or twice over aspects of economic freedom, such as property rights or investment freedom. However, others utilized an aggregate economic freedom score. Many of them discovered a favorable influence of freedom on growth. Up till now, the selection of components has been a noteworthy subject.

A sole component does not adequately describe the economic structure, and a composed index makes drawing policy implications challenging. As a result, it is critical to explore which aspects of the economic freedom indices are substantial for growth and the pathway of these impacts. So, the present study is an endeavor to examine the influence of the aggregate economic freedom index and several subcomponents of economic freedom on economic growth in four South Asian nations from 1995 to 2021. The Ordinary Least Squares, Random Effect Model, and Robust Least Squares approaches are utilized to estimate the composed and decomposed influence of economic freedom on economic growth. Robust Least Squares is also used to redirect the robustness of the linking between freedom and growth.

According to the results of these tests, economic sovereignty has a strong favorable influence on growth. In particular, economic freedom has a positive and significant impact on economic prosperity. It is treasured as an end itself and is a vigorous element of social self-esteem, sovereignty, and individual enablement. Earlier studies also reinforce the existing study. Bashir and Xu [59] perceive the positive and significant impact of political sovereignty and economic liberty on economic progress by utilizing the empirical data of 117 economies observing the period from 1980 to 2012. Equally important is the fact that economic freedom offers a tried-and-true plan for economic growth and social attainment. Esposto and Zaleski [60] specify the positive and significant impact of economic freedom on social indicators such as life expectancy and educational attainment throughout the nations of the globe.

When we evaluated the different sorts of economic liberty individually, we revealed that the coefficients of most of the sorts of economic sovereignty are significant. However, the coefficient of monetary freedom is negligible. However, the results of government disbursement, government integrity, and labor freedom are equivocal. The tax burden has a destructive and substantial influence on the economic prosperity of the countries considered. Padda and Akram [61] found the adverse impacts of a tax burden on economic growth and output in Asian countries. That burden of taxation induces companies and individuals to shift their assets to other countries and reduce the economic activities in the home markets. Ozpence and Mercan [62] revealed the opposing effects of a tax burden on economic growth in the case of Turkey. The coefficients of Property rights specify positive and significant influence on economic growth. Earlier studies accompany such significant results.

Goldsmith [27] found interesting outcomes by reviewing the relationship between property rights, democratic choices, and economic growth of 59 developing and developing countries. He found that democratic choices and property rights were positively associated with economic growth, signifying that economic progress in deprived countries is attributed to the enactment of these institutions. Sonin [63] observed comparable results in Russia, where poor and unequal countries provide fewer property rights due to bad law and order, less developmental budget, and degraded institutional eminence. Even though developed and mature economies give more property rights which cause to grow the economic and financial activities. Our results are also consistent with Cebula [64]. He examined the impact of property rights on OECD countries' economic development. Higher levels of property privileges were found to increase the rate of economic performance. Investment liberty, freedom to trade, and financial sovereignty all have an affirmative, robust, and substantial stimulus on economic growth. The results of the assenting effect of business freedom, investment sovereignty, trade liberty, and financial freedom on economic prosperity are consistent with earlier studies. Singh and Gal [65] estimated the notable and positive effect of business freedom, trade freedom, and investment freedom on FDI in Latin American, African, European, and East Asian countries. Ciftci and Durusu-Ciftci [66] perceived the noteworthy and positive effect of trade and investment freedom on economic growth and FDI in the case of OECD economies.

For 2006–2010, Haider et al. [67] looked into the relationship between business regulations and economic growth in 172 nations. He discovered that changes in business regulation had a positive and notable impact on the economies of these countries. That positive impact is because of access to energy resources, fewer environmental hazards in business, improved and well-functional business regulatory authorities, and women's economic inclusion. Gwartney et al. [28] conducted a historical analysis of the world. They found a positive correlation between financial autonomy, investment authorization, trade liberty, and economic growth. Investment and trade freedom allows individuals to move their capital to a profit-maximizing country and cause higher economic growth. Gehring [68] observed the significant and positive influence of trade liberty on the economic growth of 86 developing and developed countries of the world in the era 1990–2005. That positive impact was observed due to fewer tariff and nontariff barriers on import and export goods and services and a high trade-to-GDP ratio.

The distinct influence of each component of economic freedom will help formulate policy choices. Governments must now avoid difficulties worsened by misguided measures that distort markets, remove incentives to work and create, or otherwise jeopardize the prospects for speedy recovery and prosperity. In the long term, the most effective method to rejuvenate a society's economic life is restoring what we recognize as the best economic freedom rather than more outstanding government conduct and supervision. Besides, economic and public policies should mainly be intended to increase the level of business, financial, investment and trade freedoms.

## Declaration

### Author contributions

Shabbir Ahmed: Conceived and designed the experiments; Performed the experiments; Analyzed and interpreted the data; Wrote the paper. Mansoor Mushtaq: Conceived and designed the experiments; Performed the experiments; Analyzed and interpreted the data; Wrote the paper. Mochammad Fahlevi: Performed the experiments; Analyzed and interpreted the data; Contributed reagents, materials, analysis tools or data; Wrote the paper. Mohammed Aljuaid: Analyzed and interpreted the data; Contributed reagents, materials, analysis tools or data; Wrote the paper. Sebastian Saniuk: Analyzed and interpreted the data; Contributed reagents, materials, analysis tools or data; Wrote the paper

### Funding

The authors would like to thank King Saud University for funding this work through the Researcher Supporting Project (RSP2023R481), King Saud University, Riyadh, Saudi Arabia.

### Institutional review board statement

Not applicable.

### Informed consent statement

Not applicable.

### Data availability statement

The open-access datasets employed in the analyses can be accessed from the following links: https://databank.worldbank.org/source/world-development-indicators (accessed on May 09, 2022); https://www.rug.nl/ggdc/productivity/pwt/?lang = en (accessed on May 09, 2022); https://www.heritage.org/index/explore (accessed on May 10, 2022).

### Conflicts of interest

The authors don't have any conflict of interest.

## CRediT authorship contribution statement

**Shabbir Ahmed:** Conceptualization, Methodology, Software, Writing- Original draft preparation. **Mansoor Mushtaq:** Conceptualization, Data curation, Writing- Original draft preparation. **Mochammad Fahlevi:** Conceptualization, Methodology, Software, Supervision. **Mohammed Aljuaid:** Formal analysis, Project administration, Funding acquisition. **Sebastian Saniuk:** Writing- Reviewing and Editing, Software, Validation.

## References

[1] Alesina A., Hems L.C., Chinnock K. (1998). Annual World Bank Conference on Development Economics.

[2] Acemoglu D., Johnson S., Robinson J.A. (2001). The colonial origins of comparative development: an empirical investigation. Am. Econ. Rev..

[3] North D.C., Thomas R.P. (1973).

[4] Barro R.J. (1991). Economic growth in a cross section of countries. Q. J. Econ..

[5] Berggren N. (2003). The benefits of economic freedom: a survey. Indepen. Rev..

[6] World Bank, World Development Report 2002: Building Institutions for Markets, Oxford University Press, New York, 2002.

[7] Knack S., Keefer P. (1995). Institutions and economic performance: cross-country tests using alternative institutional measures. Econ. Polit..

[8] Bencivenga V.R., Smith B.D. (1991). Financial intermediation and endogenous growth. Rev. Econ. Stud..

[9] King R.G., Levine R. (1993). Finance and growth: schumpeter might be right. Q. J. Econ..

[10] Fedderke J. (2002). Technology, human capital, growth and institutional development. Theoria.

[11] Barro R.J. (1994).

[12] Easton S.T., Walker M.A. (1997). Income, growth, and economic freedom. Am. Econ. Rev..

[13] Fahlevi M., Aljuaid M., Saniuk S. (2022). Leadership style and hospital performance: empirical evidence from Indonesia. Front. Psychol..

[14] de Haan J., Lundstrom S., Sturm J.-E. (2006). Market-oriented institutions and policies and economic growth: a critical survey. J. Econ. Surv..

[15] Gwartney J., Lawson R., Hall J. (1999).

[16] Torstensson J. (1994). Property rights and economic growth: an empirical study. Kyklos.

[17] Arora V., Vamvakidis A. (2006). The impact of U.S. Economic growth on the rest of the world: how much does it matter?. J. Econ. Integrat..

[18] de Vanssay X., Spindler Z.A. (1994). Freedom and growth: do constitutions matter?. Publ. Choice.

[19] Scully G.W., Slottje D.J. (1991). Ranking economic liberty across countries. Publ. Choice.

[20] Clark J.R., Lawson R.A. (2008). The impact of economic growth, tax policy and economic freedom on income inequality. J. Priv. Enterprise.

[21] Cebula R.J., Mixon F.G. (2012). The impact of fiscal and other economic freedoms on economic growth: an empirical analysis. Int. Adv. Econ. Res..

[22] Mushtaq M., Ahmed S., Fahlevi M., Aljuaid M., Saniuk S. (2022). Globalization and employment nexus: moderating role of human capital. PLoS One.

[23] Smith A. (1976).

[24] Hayek F.A. (2020).

[25] Mises L. (1996).

[26] Rothbard M.N. (2000).

[27] Goldsmith A.A. (1995). Democracy, property rights and economic growth. J. Dev. Stud..

[28] Gwartney J.D., Lawson R., Hall J. (2017).

[29] Habiburrahman, Prasetyo A., Raharjo T.W., Rinawati H.S., Trisnani, Eko B.R., Wahyudiyono, Wulandari S.N., Fahlevi M., Aljuaid M., Heidler P. (2022). Determination of critical factors for success in business incubators and startups in East Java. Sustainability.

[30] Weede E., McMahon F. (2012). Towards a Worldwide Index of Human Freedom.

[31] Rindermann H. (2018).

[32] Hoppe H.-H. (2001).

[33] Nelson M.A., Singh R.D. (1998). Democracy, economic freedom, fiscal policy, and growth in LDCs: a fresh look. Econ. Dev. Cult. Change.

[34] Wu W., Davis O.A. (1999). The two freedoms, economic growth and development: an empirical study. Publ. Choice.

[35] Heckelman J.C. (2000). Economic freedom and economic growth: a short-run causal investigation. J. Appl. Econ..

[36] Heckelman J.C., Stroup M.D. (2002). Which economic freedoms contribute to growth?. Reply Kyklos..

[37] Carlsson F., Lundstrom S. (2002). Economic freedom and growth: decomposing the effects. Publ. Choice.

[38] Bengoa M., Sanchez-Robles B. (2003). Foreign direct investment, economic freedom and growth: new evidence from Latin America. Eur. J. Polit. Econ..

[39] Chheng K. (2005).

[40] Averre D. (2007). “Sovereign democracy” and Russia's relations with the European union. Demokratizatsiya.

[41] Justesen M.K. (2008). The effect of economic freedom on growth revisited: new evidence on causality from a panel of countries 1970–1999. Eur. J. Polit. Econ..

[42] Saribas H. (2010). Economic freedom and economic well-being: a granger causality analysis of 49 countries, 1995–2004. Appl. Econom. Int. Dev..

[43] Garrett T.A., Rhine R.M. (2010).

[44] Pourshahabi F., Mahmoudinia D., Soderjani E.S. (2011). FDI, human capital, economic freedom and growth in OECD countries. Research Journal of Internatıonal Studıes..

[45] Kilic C., Arica F. (2014). Economic freedom, inflation rate. Romanian J. Econ. Forecast..

[46] Kesikoglu F. (2015). Economic freedom and economic growth: a panel causality analysis for selected transition economies. The Macrotheme Review.

[47] Haydaroglu C. (2016). The effect of foreign direct investment and economic freedom on economic growth: the case of BRICS countries. Res. World Econ..

[48] Ahmad M. (2017). Economic freedom and income inequality: does political regime matter?. Economies.

[49] Medina-Moral E., Montes-Gan V.J. (2018). Economic freedom, good governance and the dynamics of development. J. Appl. Econ..

[50] Tran D.V. (2019). A study on the impact of economic freedom on economic growth in ASEAN countries. Bus. Econ. Horiz..

[51] Ahmed S., Ahmad H.K. (2020). Impact of economic and political freedom on economic growth in Asian economies. Eur. Online J. Nat. Soc. Sci..

[52] Ahmed S., Ahmad H.K. (2021). Impact of economic freedom on employment in Asian countries. Int. Rev. Soc. Sci..

[53] Agresti A. (2012).

[54] Hausman J.A. (1978). Specification tests in econometrics. Econometrica.

[55] Compton R.A., Giedeman D.C., Hoover G.A. (2011). Panel evidence on economic freedom and growth in the United States. Eur. J. Polit. Econ..

[56] Mahmood K., Azid T., Chaudhry I.S., Faridi M.Z. (2010). Impact of economic freedom on economic growth: the case of some selected SAARC member countries. International Research Journal of Finance and Economics.

[57] Paakkonen J. (2010). Economic freedom as driver of growth in transition. Econ. Syst..

[58] Hall J.C., Lawson R.A. (2014). Economic freedom of the world: an accounting of the literature. Contemp. Econ. Pol..

[59] Bashir M.F., Xu C. (2014). Impact of political freedom, economic freedom and political stability on economic growth. J. Econ. Sustain. Dev..

[60] Esposto A.G., Zaleski P.A. (1999). Economic freedom and the quality of life: an empirical analysis. Consititut. Polit. Econ..

[61] Padda I.H., Akram N. (2009). The impact of tax policies on economic growth: evidence from South-Asian economies. Pakistan Dev. Rev..

[62] Ozpence O., Mercan N. (2020). The relationship between tax burden and economic growth: Turkey case. Journal of Business Economics and Finance.

[63] Sonin K. (2003). Why the rich may favor poor protection of property rights. J. Comp. Econ..

[64] Cebula R. (2011). The Impact of property rights freedom on economic growth: evidence from the OECD nations. Int. J. Econ. Res..

[65] Singh D., Gal Z. (2020). Economic freedom and its impact on foreign direct investment: global overview. Rev. Econ. Perspect..

[66] Ciftci C., Durusu-Ciftci D. (2022). Economic freedom, foreign direct investment, and economic growth: the role of sub-components of freedom. J. Int. Trade Econ. Dev..

[67] Haider N., Khan N., Iqbal N. (2015). Impact of corporate governance on firm financial performance in islamic. Int. Lett. Soc. Humanist. Sci..

[68] Gehring K. (2013). Who benefits from economic freedom? Unraveling the effect of economic freedom on subjective well-being. World Dev..

